# Pharmacological Strategies and Recent Advancement in Nano-Drug Delivery for Targeting Asthma

**DOI:** 10.3390/life12040596

**Published:** 2022-04-18

**Authors:** Aftab Ahmad

**Affiliations:** Health Information Technology Department, Faculty of Applied Studies, King Abdulaziz University, Jeddah 21589, Saudi Arabia; aftab786sa@hotmail.com or abdulsalam@kau.edu.sa; Tel.: +966-012-6400000 (ext. 75543)

**Keywords:** asthma, nano-drug delivery, liposome, polymers, dendrimers

## Abstract

With a high prevalence globally, asthma is a severe hazard to human health, as well as an economic and social burden. There are now novel therapies available for asthma with the use of nanotechnology. Recent developments in nanoscience and medicine have encouraged the creation of inhalable nanomedicines that can enhance the efficacy, patient compliance, and life quality for sufferers of asthma. Nanocarriers for asthma therapy, including liposomes, micelles, polymers, dendrimers, and inorganics, are presented in depth in this study as well as the current research status of these nanocarriers. Aerosolized nanomaterial-based drug transport systems are currently being developed, and some examples of these systems, as well as prospective future paths, are discussed. New research subjects include nano-modification of medicines and the development of innovative nano-drugs. Clinical experiments have proven that nanocarriers are both safe and effective. Before nanotherapy can be applied in clinical practice, several obstacles must be addressed. We look at some of the most recent research discoveries in the subject of nanotechnology and asthma therapy in this article.

## 1. Introduction

**Figure 1:**
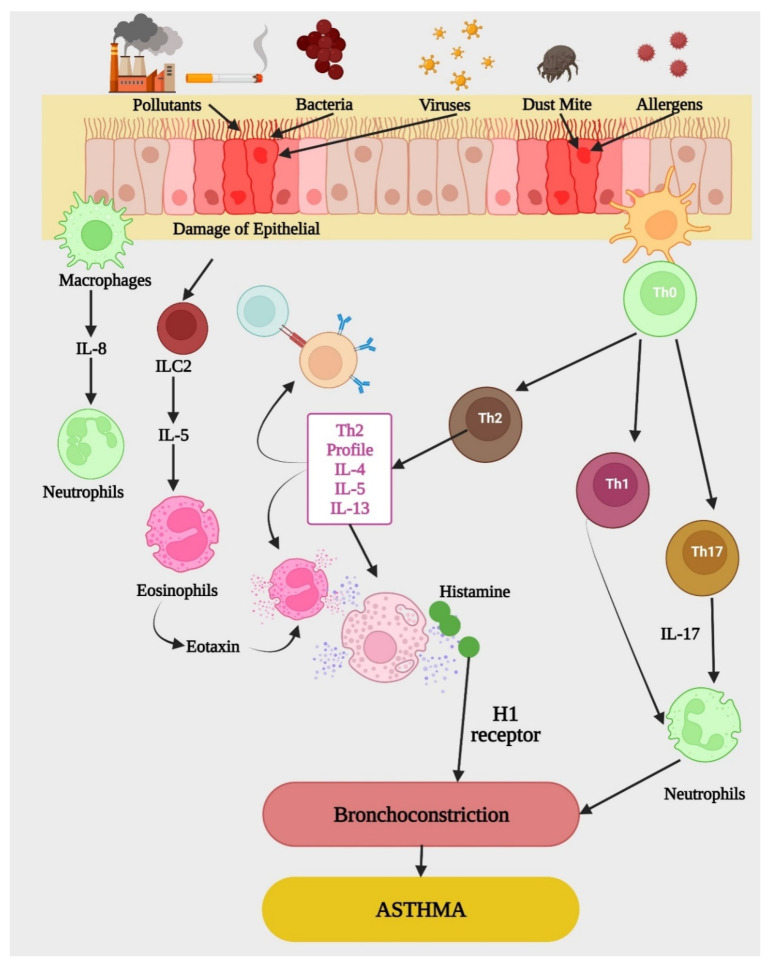
Etiopathogenesis of asthma. Cells, chemokines, and cytokines, all have a role in the development of asthma. Neutrophils and eosinophils are two types of effector cells. In the case of an allergic response mediated by IgE, eosinophils are activated, resulting in the production of cytokines such as IFN-γ, IL-13, and IL-4, as well as histamine, which is released. More research is needed to understand the neutrophilic response; however, it may represent a transition from early Th2 or the result of early Th1/Th17 activation and IL-8 release. H1, histamine; IFN-γ, interferon gamma; IgE, immunoglobulin E; IL, interleukin; ILC, innate lymphoid cell; Th, T helper cells.

Asthma is a prevalent chronic non-communicable illness in both children and adults and may be life-threatening. With an estimated global asthma population of 400 million by 2025, there are already more than 300 million individuals who suffer from the disease today. Allergies, such as asthma, are complicated ailments that are impacted by a variety of inherited and environmental factors [1,2]. Dusting, infections, smoking, weather changes, animals, perfume odors, house dust mite, hormonal changes, rain, exercise, viral infection, etc., are common triggers, and there are many distinct phenotypes based on clinical features such as the age of start and severity of illness as well as inflammatory forms (e.g., paucigranulocytic, neutrophilic, eosinophilic, and mixed granulocytic) [3]. The illness is characterized by symptoms such as bronchoconstriction generated by airway hyperresponsiveness, increased mucus secretion, and chronic inflammation, among others [4]. Asthma has no particular therapy owing to its complicated etiology, although long-term systematic treatments may successfully decrease symptoms, attacks, and enhance the prognosis [5]. Combined steroid and bronchodilator therapy such as LTRAs (leukotriene receptor antagonists) or “SABAs” (short-acting β-agonists) or “LABAs” (long-acting β-agonists) are regarded as the first-line approach for asthma management, according to the literature [6]. Even after using the highest dosage of corticosteroids, some asthmatic individuals still have poor asthma control, which is called a steroid resistance asthma. Importantly, more than 60% of asthma-related medical expenditures are incurred by these individuals [7,8]. Apart from inhaled corticoids, other treatments for moderate to severe refractory asthma include human monoclonal antibodies, cytokine/chemokine antagonists, and nebulized glucocorticoids. Although effective, these approaches are restricted by the wide variety of asthma symptoms and types. In recent years, there has been significant progress in the field of nanotechnology. According to several studies, the efficiency of anti-asthma drugs may be enhanced by the use of nano-delivery technologies [9,10] (Figure 1).

As an interdisciplinary area of study, nanotechnology focuses on the manipulation and control of sub-atomic particles and molecules with diameters between 10 nanometers (nm) and 100 nanometers (nm) [11]. As a pharmaceutical utility of nanotechnology, it can be used in a variety of ways, including for focused diagnosis and treatment, increased medication solubility and accessibility in the body, decreased drug adverse effects, and circumventing human body barriers. In addition to constructing the huge contact surface area of airways, alveolar cells and goblet cells are also involved in the formation of the initial bronchiole cells, which are composed of bronchial epithelial cells and Clara cells, respectively (mucus producing cells) [12,13]. Basement membrane is a kind of membrane that is used by both alveolar type I epithelial cells and endothelial cells in the alveolar space, and it is composed of lipids.0.1 to 0.2 microns in thickness; the air-blood barrier in the lungs is formed by a thin layer of epithelial and endothelial tissue, which is linked by the basement membrane. Because of their particular properties, the lungs, with their low barrier and high permeability, are a suitable target for both systemic and local medicine delivery. Additional advantages include improved biocompatibility and delivery of medications to lung-specific locations by pulmonary administration [14,15]. Due to the progress of nanotechnology, a new and complete perspective on respiratory disease therapy and diagnostics is now available, with the potential to improve the effect of respiratory illness treatment and diagnostics in several ways. Since its inception, nanotechnology has been a major factor in the advancement of biomedicine, with applications ranging from early illness detection and treatment to disease prevention and bioengineering research, all of which show great promise. A huge variety of nano-size materials have been utilized in biomedical research, numbering in the thousands [16,17]. In the last decade, approximately 25,000 publications have discussed nanoparticles as a medication delivery method. Cancer and infectious illnesses are the primary focus of nanomedicine. Examples include the development of intravenous anticancer medicines such as doxorubicin liposomes, paclitaxel linked to albumin, and pegaspargase. Phase II and III clinical studies are underway for more anticancer medicines. Little research, however, has been concentrated on the development of asthma therapy nanoparticles [18,19,20]. In this review, we looked into the most recent developments in this area and their use for the management of asthma patients.

## 2. Selection of Literature

Medline, Mendeley, Science Direct, Google Scholar, PubMed, and the Springer link were used to find the appropriate results at a technical level. The literature review includes a large number of terms, both individually and collectively. Some of the keywords for literature evaluation are “asthma”, “epidemiology and etiology of asthma”, “pathophysiology asthma”, “nanotechnology”, “liposomes in asthma”, “polymeric nanoparticles in asthma”, “nano-dendrimers in asthma”, “nano-micelles in asthma”, “nano-emulsion in asthma”, “gold nanoparticles in asthma”, “silver nanoparticles in asthma”, “carbon nanotubes in asthma”. We included only studies from research papers published in the English language. A reference list of relevant articles was also checked, even if they were not found via the original search technique.

## 3. Nano Drug Delivery for Targeting Asthma

**Figure 2:**
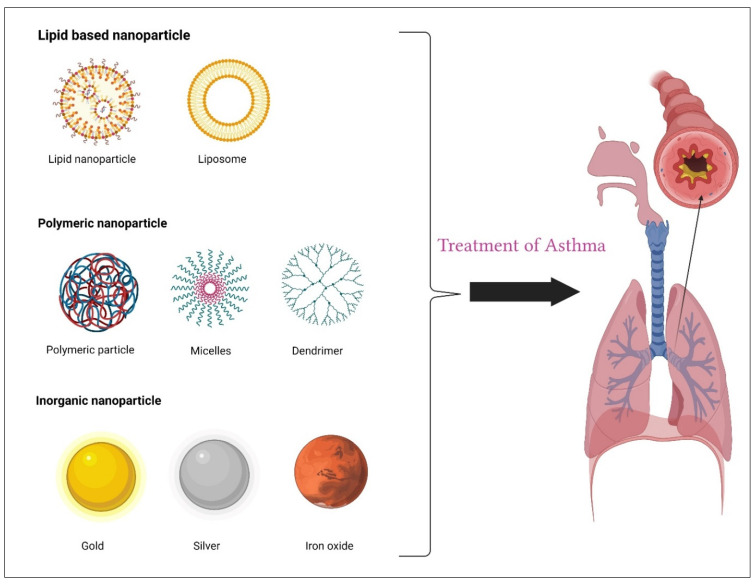
Classes of nanoparticles for the treatment of asthma.

The delivery of drugs specifically to the lungs seems to be a potential treatment approach. To put it another way, this is due to the particular anatomical structure of the lungs, which includes attributes like their small epithelial barrier thickness and their large alveolar-region surface area as well as their high degree of vascularization and low proteolytic activity [21]. Lung-targeted medication delivery techniques have been shown to benefit lung illnesses including asthma, and they may even boost the therapeutic efficacy [22]. Because the medicine may be administered intravenously or intratracheally, pulmonary delivery is straightforward. Recent advances in science and technology have made nanotechnology a viable platform for improved illness detection and therapy [23,24]. There are several benefits that nanocarriers offer over standard drug delivery systems when it concerns pharmacokinetics and immunogenicity [25]. Developing artificial nanoparticles (NPs) may be a viable way to overcome these significant standard therapeutic challenges [26,27]. NPs are primarily divided into two groups based on their chemical composition, namely organic NPs and inorganic NPs. Drugs and antibodies may be delivered using NPs as delivery vehicles. The organic NPs include liposomes, polymeric NPs, dendrimers, and micelles whereas inorganic includes iron oxide NPs, gold NPs, silica NPs, quantum dots, graphene oxide, and carbon nanotubes [25,28]. Here we discuss some of the NPs (Figure 2).

## 4. Lipid-Based Nanoparticle

### 4.1. Liposomal NPs

Liposomes have piqued attention as a potential substitute for oral administration for treating respiratory disorders like asthma since they are noninvasive and offer a long-lasting medication release. Using liposome-based medication transport to the lungs may increase drug retention, resulting in fewer extra-pulmonary adverse effects and greater therapeutic effectiveness in the therapy of people with asthma. They were offered as a novel strategy for effective asthma therapy because of the feasibility, biocompatibility, and advantages of the liposomal formulation over the currently available conventional formulations [29,30].

Salbutamol sulphate nanoliposomal dry powder inhalation for asthma treatment was developed by Honmane, et al. [31]. In this study, liposomes were synthesized utilizing the thin film hydration process and the formulation was adjusted based on vesicle size and liposome drug entrapment percentage, respectively. The optimized liposomal formulation had a mean diameter of 167.2 ± 0.170 nm. When it comes to treating asthma and other respiratory disorders, the novel liposomal formulation might be a helpful alternative to traditional treatments [32]. The reversed-phase evaporation approach was used by Arafa, et al. to create niosomes that contain salbutamol sulfate [33]. These were spherical niosomes with 400–451 nm particles that contained 66.29% of salbutamol sulfate, and the release of the salbutamol sulfate from the niosomes had an 8-h controlled release profile, showing 76.54 ± 0.132% SS concentration in solution. According to the findings, entrapping drugs in niosomes which can be bundled into aerosols meeting USP rules, this is a promising option for developing a controlled drug delivery system [34]. Using an aerosolized liposome formulation, Chen and colleagues were able to deliver anti-asthmatic medication to the lungs while also investigating the relationship between the formulation’s bioavailability and anti-asthmatic efficacy [35]. Salbutamol sulfate (SBS) was chosen as the model drug in this study because of its high-water solubility and fast absorption after administration. It has been shown that liposome-encapsulated salbutamol sulfate works well in the treatment of asthma. Liposome encapsulation of salbutamol sulfate was shown to be 70 percent efficient. The liposome suspension of salbutamol sulfate has a particle size of 57 ± 21 nm. Using a rat model, researchers were able to successfully transfer liposomes to the respiratory system and lungs by pulmonary administration [35]. The release of salbutamol sulfate from liposomes remained for at least 48 h after administration. Liposomes increased salbutamol sulfate concentration and retention duration, and hence prolonged the salbutamol therapeutic impact on asthma patients [35]. Salbutamol sulfate and beclometasone dipropionate were combined in tiny unilamellar liposome vesicles by Elhissi, et al. Rehydrated liposomes containing salbutamol sulfate and beclomethasone dipropionate had the lowest sizes when made from sucrose or trehalose, with sizes below 100 nm and below 136 nm, respectively. The results of this investigation showed that freeze-dried liposomes containing two anti-asthma medicines could be produced and that they could be used in pulmonary delivery [4,36]. Konduri, et al. determined whether weekly therapy with budesonide encapsulated in sterically stabilized (stealth) liposomes would be comparable to daily budesonide therapy in reducing allergic inflammation. Ovalbumin-sensitized C57/Black 6 mice received aerosolized (1) budesonide encapsulated in stealth or conventional liposomes, administered weekly, (2) budesonide (without liposomes), administered either daily or weekly, or (3) empty stealth liposomes, administered weekly. All treatment groups were compared with sensitized untreated or unsensitized mice. The result indicated that weekly therapy with budesonide encapsulated in stealth liposomes was as effective as daily budesonide therapy in decreasing lung inflammation and lowering eosinophil peroxidase activity, peripheral blood eosinophils, and total serum IgE levels [37].

Curcumin and salbutamol, loaded together in liposomes were prepared by Ng et al. utilizing the lipid hydration technique. With an average diameter of 271.3 ± 3.06 nm, the produced curcumin formulation also had a very low zeta potential, measuring just −61.0 mV. Comparing the two formulations of curcumin-loaded liposomes to the LPS administered group, both formulations showed substantial (*p* < 0.05) reductions in the secretion of proinflammatory biomarkers like, interleukin (IL)-1b, IL-6, IL-8, and tumor necrosis factor-alpha (TNF-α) expression. Liposomal curcumin may be an asthma therapeutic strategy because of its ability to decrease a key asthma pathogenesis pro-inflammatory signal [38]. Liposomes encapsulating allergens and CpG were shown to effectively alleviate allergic asthma symptoms when given for brief periods by Alberca-Custodio and colleagues [39]. Researchers investigated the possibility of using immunotherapy to treat a relevant respiratory allergen derived from *B. tropicalis* mite extract, which is found in some of the most prevalent tropical countries. They discovered that treatments with co-encapsulated *B. tropicalis* allergens and CpG were effective in reversing the already established allergic lung responses in asthma patients. The immunotherapy liposomal delivery approach may be beneficial in the treatment of eosinophilic (high type 2) asthma endotype, and may hint at the crucial role performed by dendritic cells expressing Myd88 in the process [39]. Mesalamine-loaded liposomes in disease-responsive microgels were developed by Raju, et al. to treat allergic asthma. Raw 264.7 cell line was used to evaluate liposomes and microgels for expression of janus kinase (JNK), P38 map kinase, and nuclear factor kappa B (NF-κB). This study found that the formulation-treated group had a two-fold decrease in the expression of nuclear factor-kappa B. P38 map kinase while JNK expression levels were found to be similar [40]. Liposome-entrapped *D. pteronyssinus* immunization in moderate asthma patients was studied by Alvarez, et al. in clinical trials. A double-blind, placebo-controlled trial was conducted on 26 asthma patients who randomly received vaccination or placebo for 1 year. The levels of exposure to Der p 1 allergen were constant during the study. Allergen bronchial challenge was made at the beginning (T0) and after 1 year of treatment (T12). The day before and 24 h after the allergen provocation, patients were challenged with methacholine (Mth) (until FEV1 fell by 40%) and blood and sputum samples were obtained. Dose-response curves to Mth were evaluated in terms of Mth-PD20 (dose of Mth that induced 20% drop in FEV1), slope (Mth-DRS), and level of plateau. Blood and sputum eosinophils, serum levels of eosinophil cationic protein (ECP), and intercellular adhesion molecule-1 (ICAM-1) were measured. Liposome-entrapped *D. Pteronyssinus* immunization has been shown to protect moderate asthma patients from exacerbation of asthma due to prolonged mite exposure, and to diminish allergen bronchial provocation-induced functional and inflammatory alterations [41]. The anti-asthmatic effects of nebulized R-terbutaline hydrochloride liposome were evaluated in guinea pigs by Li, et al. This study used an ammonium sulphate produced transmembrane electrochemical gradient to load R-terbutaline hydrochloride into liposomes. A particle size of 145 ± 20 nm was obtained using this approach. The R-terbutaline hydrochloride liposome group exhibited a longer anti-asthma effect than the R-terbutaline hydrochloride solution group [42]. For the therapy of persistent asthma and chronic obstructive pulmonary disease (COPD), Arora, et al. developed a double-hydration liposomal dry powder inhaler with doxophylline. The gamma scintigraphy in vivo investigation found that doxophylline was better retained in the liposomal formulation than in the controlled release formulation, as was shown by the results [43] (Table 1).

### 4.2. Lipid Nanoparticles

For a long time, solid lipid nanoparticles (SLN) have been widely studied for the delivery of pulmonary medication. SLNs are triglyceride and phospholipid-rich aqueous nanoscale suspensions. Toxicologically, these formulations are less harmful, making them more suited for inhalational administration of pharmaceuticals [45]. The deep lungs are rich in phospholipids, which are necessary for the respiratory process to work. For optimal surface tension and reduced lung tissue friction, phospholipid-based surfactant proteins must be present on the alveolar surface. A 30:70 SLN phospholipid/triglyceride ratio was shown by Nassimi and colleagues for pulmonary delivery [46]. Quercetin-loaded solid-lipid micro-particles (SLM,) were recently reported for physico-chemical investigation and examined for possible application in asthma therapy due to their antioxidant and anti-inflammatory characteristics of flavonoid quercetin One of these quercetin-SLMs was made by synthesizing glyceryl trimethyl silane and soy lecithin and achieving an appropriate mean mass aerodynamic diameter (MMAD) [47,48]. Particles were shown to be stable during nebulization and deposited mostly in the deep lung areas, according to in vitro deposition tests [48]. Curcumin was added to a stearic acid and lecithin SLN produced by Wang, et al. in the second research. The solvent injection was used to generate SLN loaded with curcumin for the treatment of asthma. This was shown to be stable in a diameter range of 190 to 200 nm. The curcumin-treated group was shown to have lower cytokine levels compared to the untreated group in an in vivo experiment involving ovalbumin-induced asthma. Furthermore, curcumin-SLN reduced airway hyperresponsiveness and inflammatory cell infiltration, as well as the lower production of cytokines, including interleukin-4 and interleukin-13, indicating that it may be useful in the treatment of asthma. Chuanfeng et al. developed rhynchophylline SLNs to boost their pharmacological efficacy in a mouse allergy experimental asthma model in another investigation. In mouse experimental asthma, he discovered the antioxidative activity of rhynchophylline-SLNs induced by ovalbumin. The rhynchophylline-SLNs also inhibited the remodeling of the airway better (including mucus gland hyper-plasia and collagen deposition) as compared with rhynchophyllin (Rhy). The authors discovered that Rhy-SLNs alleviated allergic asthma by upregulating the level of cytokine signaling-1 and suppressing the p-38 signaling-pathways [49].

## 5. Polymeric Based Nanoparticle

### 5.1. Nano Polymeric Particles

Experimental allergic asthma is prevented from remodeling by thymulin gene therapy delivered by DNA nanoparticles, according to A.L. da Silva and coworkers. Improved lung mechanics were shown when a single dose of thymulin-plasmid-carrying DNA nanoparticles was given to a murine model with ovalbumin-challenged allergic asthma. This treatment reduced inflammation, collagen deposition, and smooth muscle hypertrophy. Researchers were encouraged by the results to continue working on nanoparticle-based gene treatments to deliver therapeutic genes for asthma safely and effectively [50,51]. Chitosan–hyaluronic acid nanoparticles coated with heparin were produced by Oyarzun-Ampuero, et al. for the treatment of asthma. Microscopic examination indicated that mast cells had taken up the nanoparticles of heparin-loaded chitosan–hyaluronic acid. However, the ability of free heparin to suppress histamine release and that of heparin encapsulated in the nanosystems to do so was extremely comparable and exhibited the same dose-response dependency [51]. An aerosolized, ferulic acid-loaded chitosan nanoparticle was created by Dhayanandamoorthy, et al. employing a vibrating mesh nebulizer as a strategic combination of the medication, nanocarrier, and delivery mechanism for successful asthma treatment. It was shown that hyaluronic acid functionalization increased the thermal stability and therapeutic efficiency of ferulic acid by enabling greater interaction with and transportation through the mucus barrier; without this, ferulic acid has limited bioavailability and quick metabolism [20]. Atropine-loaded nanoparticles were produced by Chattopadhyay, et al., and tested on adult Wistar rats for their effects on lung hyperresponsiveness, obstruction, and inflammation induced by ovalbumin. Inflammatory cytokines, shallow breathing, and abnormally large tidal volumes were all decreased by the atropine-loaded nanoparticle. The 18-day atropine-loaded nanoparticle therapy declined collagen deposition and also the progressing blockage of the airway in the Wistar rats. Because atropine-loaded nanoparticles are given to the lungs as a nanoparticle, it enhances the lung airway surfaces, reducing hyperresponsiveness and inflammation [52]. Liquid crystal nanoparticles containing quercetin were produced and examined for their anti-inflammatory characteristics in human primary airway epithelial cell lines activated by lipopolysaccharide, and also for their ability to reduce inflammation. For asthma, quercetin-loaded and surface-modified liquid crystalline nanoparticles may be a viable treatment option because of their ability to decrease the generation of pro-inflammatory cytokine (IL-8, IL-1β and IL-6) connected to the illness [53].

Asthma-induced airway inflammation can be prevented by nanoparticle administration of anti-inflammatory LNA oligonucleotides, according to Ramelli, et al. Oligonucleotide therapies reduced the gene expression that is elevated in both the animal model of disease and human asthma. However, both oligonucleotides greatly increased the expression of several interferon signaling genes. As a consequence of these findings, locked nucleic acid/DNA oligonucleotides may be delivered I.V. and have an impact on lung inflammation, which suggests that interferon response pathways may be normalized [54]. The allergic asthma mouse model induced by OVA sensitization was used to evaluate the effects of baicalein-encapsulated/loaded chitosan nanoparticles by D. Wang, et al. Asthma’s immune-allergy-inflammatory response is controlled by loaded and encapsulated baicalein nanoparticles. Encapsulated baicalein nanoparticles both reduced airway hyperresponsiveness and inflammation through NF-κB, and AP-1 are important transcriptional processes, making them potential anti-asthma drugs [55]. PEG-PLGA nanoparticles containing bavachinin have been evaluated in a murine model induced by OVA sensitization of asthma therapy. Through oral administration, these NPs showed very good anti-asthma therapeutic effects in a murine allergic asthma model, as assessed by analysis of histological sections, local and systemic cytokine expression, and T cell differentiation. Bavachinin is an asthma medication, and researchers believe that this approach might also be employed for the oral administration of other medications with low pharmacokinetic effectiveness [56]. The anti-asthmatic qualities of andrographolide (AG), in comparison with glucocorticoids, are enhanced by the fact that it is less toxic and has fewer adverse effects. Its bioavailability, on the other hand, is lower, and its plasma half-life is shorter than that of glucocorticoids. Some researchers have attempted to address these shortcomings by encapsulating AG in nanoparticles (AGNP) for use in asthma therapy. A mouse asthma model was given oral/pulmonary administration of AG nanoformulation, and the bioavailability of the drug was greatly enhanced, as was the release of inflammatory factors (such as IL-4, IL-5 and IL-13), which were also significantly reduced. The outcomes of the study also revealed that the pulmonary route of administration has a larger therapeutic potential than oral administration in terms of efficiency and effectiveness. AGNP reduced the elevated IL-4, IL-5, and IL-13 levels significantly by the pulmonary route rather than by the oral route [57].

### 5.2. Nanosuspension

Ovalbumin-induced asthma can be efficiently treated using a drug delivery system of self-nano emulsifying which was developed by Cao et al. For the treatment of asthma, isoliquiritigenin drug delivery system of self-nano emulsifying was shown to have a much better bioavailability and anti-asthma effect than isoliquiritigenin suspensions [58]. Curcumin has shown a potential extraordinary activity as an add-on ingredient in asthma treatment, due to its immunomodulatory and anti-inflammatory mechanism of action. However, its low water solubility and bioavailability lead to a poor therapeutic effect, which can be overcome by its formulation as nanocrystals. Luca, et al., prepared a multicomponent formulation for the delivery of curcumin (CUR) and beclomethasone dipropionate (BDP) into the lungs as water-based nanosuspensions (NS). Luca and his colleagues prepared three formulations exhibiting a nanocrystal mean diameter in the range of 200–240 nm and a homogenous particle size distribution. Aggregation or sedimentation phenomena were not observed in the multicomponent formulation on 90 day storage at room temperature. Finally, the nebulization tests of the three samples showed optimal aerodynamic parameters and MMAD < 5 µm [59].

### 5.3. Nano Micelles

Intranasal micellar curcumin was designed by Chawla et al. for the therapy of persistent asthma. Curcumin-micelles (about 57.6%) and dexamethasone (approximately 59.3%) both reduced intracellular ROS levels to comparable degrees. The release of nitric oxide was also significantly inhibited by micellar curcumin, which was shown to be effective [60,61]. As a pulmonary delivery method, Sahib, et al. used polyethylene glycol–diacyl lipid micelles to contain beclomethasone dipropionate (BDP) loaded sterically stabilized phospholipid nanomicelles (SSMs). The BDP-SSMs showed a prolonged dissolution profile of about 3 days. Intratracheal administration of the BDP-SSMs (1 mg/kg) 12 or 23 h before a challenge in the asthmatic rat model led to a significant reduction in the inflammatory cell counts in bronchoalveolar lavage fluid samples compared with the administration of solubilized BDP [62]. Targeted delivery of Chil3 and Chil4 siRNA to activated macrophages was developed using a ligand called high mobility group (HMG) and oligoarginine (OR) micelles, according to Choi et al. This is found on the surface of macrophages which are activated, where HMG ligates to toll-like receptor 4 (TLR4) and receptor for advanced glycation end-product (RAGE) receptors. According to results from in vitro transfections, the triplex complexes carried siRNA, to TLR4-expressing macrophages with high specificity. The administration of siRNA/HMG/OR triplex complexes by the intra-tracheal route selectively targeted alveolar macrophages for Chil3 and Chil4 siRNAs. While Chil4 and Chil3 expression was decreased using ternary complexes, asthma symptoms such as airway irritation and mucus production were reduced [63,64,65]. Onoue et al. developed a self-assembled micellar version of chafuroside A that had improved anti-inflammatory activities (through recovering an increased release of macrophages, neutrophiles and eosinophils) in asthma model rats. In decreasing ovalbumin-induced airway inflammation, a self-assembled micellar formulation at 0.1 mg/kg was as efficient as crystalline chafuroside A at 1.0 mg/kg, suggesting that the better dissolving characteristic of the self-assembled micellar formulation chafuroside A has a stronger pharmacological impact. These findings suggest that the chafuroside A self-assembled micellar formulation for the treatment of asthma might be an effective method [65].

### 5.4. Dendrimer

Inapagolla et al. studied the ability of MP–polyamidoamine (PAMAM) dendrimer conjugate to improve airway delivery and was evaluated in a pulmonary inflammatory murine model based on an 11-fold enhancement of eosinophil lung accumulation following five daily inhalation exposures of sensitized mice to the experimental allergen, ovalbumin. For both airways and tissues, eosinophil recoveries were reduced by 71% and 47% with methylprednisolone alone. The methylprednisolone–dendrimer combination reduced these levels by 87 percent and 67 percent, respectively, compared to the same daily methylprednisolone dose. Dendrimer-conjugated methylprednisolone has been shown to boost the capacity of methylprednisolone to reduce inflammation induced by allergens, perhaps by increasing the retention time of the drug in the lung [60,66,67].

### 5.5. Miscellaneous

Vij at al., formulated an inhalation of PEGylated immuno-coupled PLGA nano-particle (PINPs) technology which was suitable to selectively target neutrophils and control inflammatory reactions related to CPDs such as asthma by targeting neutrophils. The emulsification of the DSPE-PEG-2000 along with PLGA mixed in either Nile red or ibuprofen (IBF) and incubation with NIMP-R14 anti-bodies intended for targeting neutrophil yielded PINP-NIMP-IBF-NPs, roughly of size 344 nm. PINPs targeting neutrophils was tested in mice (C57BL/6) by intra-tracheally treating them with *Pseudomonas aeruginosa* lipo-polysaccharide (Pa-LPS,) 12 h before instilling PINP-NIMP-IBF to produce pulmonary/airway inflammation and then specifically binding and releasing medication to neutrophils. Treatment with the PINP-NIMP-IBF reduced the amount of NIMP-R14_+_ BALF cells and the activity of NF-κB, after administration, demonstrating that it can control the inflammatory response. After administration, the number of NIMP-R14_+_ BALF cells and the NF-κB expression were measured. It is important to conduct further research on the bio-distribution of PINPs in the lung tissues; but this undertaken model can be used as an inhalation therapy system to treat obstructive pulmonary disorders [68]. NPs may effectively transfer therapeutic medications to damaged tissues, increase the accumulation of pharmaceuticals in the lungs, and therefore boost the therapeutic efficacy of the treatment. There is an increased accumulation of beclomethasone dipropionate in the lung, improved bioavailability, and reduced dose and adverse effects with PEGylated PAMAM dendrimer delivery. Nanocarriers transporting dexamethasone to the lung proved to be an efficient means of alleviating allergic inflammation of the lung, lowering the infiltration of eosinophils, and limiting the release of inflammation-inducing mediators, as shown in this research. Dexamethasone NPs are superior to free dexamethasone in reducing airway hyperresponsiveness [69,70].

Montelukast-loaded nanostructured lipid carriers (MNCs) were designed by Patil and colleagues to boost lung retention and minimize the cytotoxicity of strong asthma medication. One of the most often used cLTRA antidotes for asthma patients is the cysteinyl leukotriene receptor antagonist, montelukast (MTK). MNCs with a Capryolto Precirol ratio of 3:7 were prepared using melt-emulsification-ultrasonication procedures of homogenization, which resulted in particles with a diameter of around 185 nm. Mannitol was used as a cryoprotectant and a carrier in the lyophilization of MNLC-DPI particles for DPI. A549 human lung adenocarcinoma cells were subjected to an in vitro cytotoxicity assay, which demonstrated that MNLCs were more viable than the free drug. Wistar rats were given MNLCs intratracheally to test biodistribution, and the results showed that the MNLC system provided better bioavailability and a longer residence time for MTK than did the administration of free MTK in an in vivo asthma model; lipidic nanoparticle formulations boost the retention of lung MTK, while decreasing the toxic effects, that can result in an additional successful asthma therapy using MNLC-DPIs [71,72] (Table 2).

## 6. Inorganic NPs

### 6.1. Nano-Gold Particles

As one of the most commonly utilized nanoparticles, gold has numerous medical applications, including diagnosing, therapeutic, and imaging procedures. This is due to its unique properties such as a significant contact surface area compared to its volume. Gold nanoparticles offer several benefits over other nanoparticles, including their neutral nature, stability, high diffusion property, non-toxicity, environmental friendliness, and capacity to change their optical properties. According to Omlor, et al., 5 nm PEGylated and citrated gold nanoparticles had local impacts on airway inflammation as well as systemic absorption in asthmatic mice. Particularly effective are citrated gold nanoparticles, which reduce both inflammatory infiltrates and airway hyperreactivity. This study was further supported by Nasab et al. who achieved the optimal size of gold nanoparticles (100–500 nm) as anti-asthma drugs and peptide carriers. This modified and manipulated method can be useful because of low cost and can be carried out in minimum time [73]. However, while developing and evaluating asthma medicines based on gold nanoparticles, it is important to examine their systemic absorption and the consequences of this uptake, as well as the potential for deleterious effects on the immune system [74,75].

### 6.2. Nano-Silver Particles

As a nanotherapeutic system, silver has lately acquired popularity because of its cytoprotective and healing properties, which make it a generally safe and hypoallergenic metal [76]. Using AgNPs, Jung and his colleagues showed that they could reduce hyper-responsiveness and bronchial inflammation, both of which are frequent in CPDs. AgNPs were dissolved in PBS and solubilized AgNPs were designed with a diameter of approximately 6 nm. Laboratory mice exposed to OVA commonly used allergens that cause asthmatic pulmonary inflammation received daily inhalations of silver NPs by jet nebulization for five days to test the NP anti-inflammatory effects. To test this, they took samples of BAL and discovered that AgNPs had lowered the numbers of inflammatory cells and indicators (neutrophils, eosinophils, and lymphocytes) that are associated with airway hyper-responsiveness and asthma. There was also a reduction in the production of Th2 cytokines (IL-4 and IL-5), a key indicator of asthma-inducing immunology. Interestingly, the enhanced intracellular ROS levels in BAL fluid collected following the OVA challenge were much lower after the injection of AgNPs, suggesting that AgNPs are capable of attenuating the effects of oxidative stress, bronchial hyperreactivity, and smooth muscle precontraction. Because of their antioxidant and anti-inflammatory effects, AgNPs seems to hold promise as an inhalation treatment for asthma [77,78,79].

### 6.3. Iron Oxide Nanoparticles

Magnetic materials have been intensively investigated for application in drug delivery systems due to their imaging and therapeutic properties. To control asthmatic inflammation, researchers developed antibody-conjugated, polymer-coated superparamagnetic iron oxide nanoparticles (SPIO NP), which block the inflammatory pathway activated by the immune system’s interleukin-4 receptor (IL4R) [80]. SPIO NPs have minimal inherent toxicity, are easily functionalized on the surface, and maybe easily identified by MRI. IL4R-nanoparticles are made up of an anti-IL4R SPIO nanoparticle with a dextran shell, which is coupled to polyethylene glycol (PEG2000) chains, and which has been conjugated to anti-IL4R monoclonal antibodies (anti-IL4R-NPs). These nanoparticles have a diameter of 133 nm. Anti-IL4Ra-NPs were administered intravenously to BALB/c OVA-sensitized mice using a micro sprayer aerosolizer. The capacity of CD8+ and CD4+ T cells to generate proinflammatory cytokines was significantly reduced by anti-IL4R NPs as well as the free antibody. Immunohistological labeling indicated the presence of anti-IL4RNPs in lung tissue regions abundant in inflammatory cells. Compared to the therapy with free anti-IL4R antibodies, they also caused less lung inflammation and kept it low for a week after the last installation. After delivery of anti-IL4 antibodies, this NP system improved lung tissue penetration, resulting in long-lasting antiinflammation benefits without the usage of corticosteroids [59,81,82,83].

### 6.4. Nano Vaccine for Asthma

In addition, nanoparticle-based immunotherapy against particular antigens that cause asthma is a potential area of research. Researchers have discovered a way to prevent and cure dust mite allergies by using a vaccine based on nanoparticles. It was shown in these investigations that when it came to the prevention of home dust mite-induced allergies, the size of nanoparticles utilized for immunization had a significant influence. Zhao, et al. studied the effect of certain Ag-guiding exosomes on reducing neutrophil-dominated airway inflammation. Exosomes containing a combination of specific Ag/anti-CD64 Ab and Fas ligand (tExo) were designed to treat neutrophilic asthma in mice by targeting specific PMN (sPMN). sPMNs were the primary cell type in BALF from ovalbumin-induced allergic asthmatic mice, whereas fewer than 3% of sPMNs were seen in naïve control mice. When activated with Ag, sPMNs produced a higher quantity of CD64, which formed complexes with Ag-specific IgG. (sIgG). sPMNs that carry the sIgG/CD64 complex may become activated in the presence of specific Ags [80]. In animal experiments, tExos had a significant impact on asthma control. sPMNs had been found in the airways of asthmatic mice. To activate the sPMNs, a particular Ag may be exposed to it. tExos can trigger apoptosis in sPMNs, indicating that it might be used to treat asthma [84,85]. After 15 weeks of immunization, Conde, et al. showed that their combination vaccines against mouse IL-13 and IL-4 are effective in lowering IgE levels in mice as well as mucus formation, eosinophilia, and AHR in preclinical models of asthma. Additionally, authors tested vaccinations specific for human IL-13/IL-4 in mice activation of the homologous receptor, interleukin (IL)-4 receptor alpha (IL-4Rα) and found that both cytokines were effectively neutralized and lowered IgE levels were maintained for at least 11 weeks after immunization. The results suggest that a long-term, cost-effective therapy for allergic asthma with a combination of IL-4 and IL-13 vaccinations may be possible, but more research is needed to ensure its safety and efficacy [86,87]. The signaling pathways mediated by chemokines and the CC chemokine receptor 3 (CCR3) are important for the discovery of asthma medications. The researchers developed a new peptide NP CCR3 inhibitor (R321) that may inhibit the signal transduction of the CCR3 receptor. Eosinophils in the airway, lung, and blood were prevented from accumulating in asthmatic mice while R321 also prevented the beginning of airway hyperresponsiveness in asthmatic mice [63,88]. Therapy of genes has played an essential part in the therapy of a variety of disorders in recent years, and it has emerged as one of the most talked-about issues in the medical community today. The use of gene therapy in conjunction with nanotechnology may prove to be a safer treatment for asthma. Chitosan-IFN–pDNA NPs (CIN) has been proven to significantly reduce allergic asthma in mice exposed to ovalbumin via chitosan-IFN-pDNA NPs (OVA) [89]. CD8+ T lymphocytes specific to OVA and dendritic cells can both be protected against pro-inflammatory chemical production by CIN, according to a new study. Asthma patients may benefit from the CIN ability to modulate T helper type 1 and type 2 immunoregulatory systems, according to the researchers [90,91].

## 7. Conclusions and Future Perspective

Numerous advantages have already been established in the administration of drugs and vaccinations using nanotechnology for asthma treatment. There are many different types of cells and components affected by asthma, which is a long-term, chronic inflammatory illness. Thus, asthma provides a wide range of possible molecular targets that might combine with nanoparticles to give drug efficacy. In order to overcome the shortcomings of pharmaceuticals, nanotechnology has become an essential weapon in the fight against drug resistance. The benefits and uses of NPs as drug delivery vehicles in asthma were discussed in this study. The use of nanotechnology in conjunction with inhaled delivery has boosted the development of asthma medications. Many studies are still in their infancy and need to be examined for their clinical impact, despite promising preclinical results. In the future, researchers should focus on therapeutic nanomedicine, molecular mechanism, performance modification, and probable toxicology.

## Figures and Tables

**Table 1:** Liposomal nanoparticle used in the treatment of asthma.

Author	Drug	Developmental Stage	Average Particle Size	Findings	Ref.
Honmane, et al.	Salbutamol sulfate	In vitro	167.2 ± 0.170 nm	The novel liposomal formulation might be a helpful alternative to traditional treatments	[31]
Arafa, et al.	Salbutamol sulfate	Phase I	400–451 nm	The entrapping drugs in niosomes, which can be bundled into aerosols meeting USP rules, is a promising option for developing a controlled drug delivery system.	[33]
Chen, et al.	Salbutamol sulfate	Preclinical studies	57 ± 21 nm	Liposomes increased salbutamol sulfate concentration and retention duration, and hence prolonged salbutamol therapeutic impact on asthma patients.	[35]
Elhissi, et al.	Salbutamol sulphate and Beclometasonedipropionate	In vitro	100–136 nm	The freeze-dried liposomes containing two anti-asthma medicines could be produced and may be used in pulmonary delivery.	[44]
Konduri, et al.	Budesonide	Preclinical studies		Minimize toxicity and increase compliance in asthma patients.	[37]
Ng, et al.	Curcumin	In vitro	271.3 ± 3.06 nm	Liposomal curcumin may be a viable asthma therapeutic strategy because of its ability to decrease key asthma pathogenesis pro-inflammatory signals.	[38]
Alberca-Custodio, et al.	Allergens and CpG	Preclinical studies		To treat eosinophilic (high type 2) asthma endotype, immunotherapy liposomal delivery method may be of benefit, and this may point to the critical role played by dendritic cells expressing Myd88 in the process.	[39]
Raju, et al.	Mesalamine	In vitro	200 nm	Mesalamine-loaded liposomes in disease-responsive microgels effective in asthmatic treatment.	[40]
Alvarez, et al.	*D. pteronyssinus*	Clinical studies		Extensive *D. Pteronyssinus* immunisation with liposome-entrapped mites protects moderate asthma patients against protracted mite exposure and lowers allergen-related functional and inflammatory alterations.	[41]
Li, et al.	R-terbutaline hydrochloride	Preclinical Studies	145 ± 20 nm	The R-terbutaline hydrochloride liposome group exhibited a longer anti-asthma effect than the R-terbutaline hydrochloride solution group	[42]
Arora, et al.	Doxophylline	In vitro	212.9 + 7.2 nm	Better retained in the liposomal formulation than in the controlled release formulation.	[43]

**Table 2:** Nanoparticles used in the treatment of asthma.

Author	Nanoparticle Type	Drug	Developmental Stage	Finding	Ref.
A.L. da Silva, et al.	Polymeric	Thymulin	Preclinical studies	Nanoparticle-based gene treatments to deliver therapeutic genes for asthma safely and effectively.	[50]
Oyarzun-Ampuero, et al.	Polymeric	Heparin	Preclinical studies	Chitosan (CS) and hyaluronic acid (HA)mucoadhesive nanocarriers and containing the macromolecular drug heparin, suitable for pulmonary delivery	[51]
Chattopadhyay, et al.	Polymeric	Atropine	Preclinical studies	Enhances the lung airway surfaces, reducing hyperresponsiveness and inflammation.	[52]
D.O. Cherk Yong, et al.	Polymeric	Quercetin	In vitro	Effective in reducing the generation of main pro-inflammatory cytokines associated with the progression of asthma.	[53]
Ramelli, et al.	Polymeric	Nucleic acid/DNA oligonucleotides	Preclinical studies	Locked nucleic acid/DNA oligonucleotides may be delivered intravenously and have an impact on lung inflammation.	[54]
D. Wang, E. MehrabiNasab, and S.S. Athari	Polymeric	Baicalein	Preclinical studies	Encapsulated and loaded Baicalein nanoparticles both reduced airway hyperresponsiveness and inflammation, making them potential anti-asthma drugs.	[55]
Wang, et al.	Polymeric	Bavachinin	Preclinical studies	Pharmacokinetically effective and rational therapy.	[56]
Chakraborty, et al.	Polymeric	Andrographolide	Preclinical studies	Nanoparticle formulations have higher bioavailability, efficacy, and efficiency than other formulations.	[57]
Cao, et al.	Nano suspension	Isoliquiritigenin	Preclinical studies	Isoliquiritigenin self-nano emulsifying drug delivery system was shown to have much better bioavailability and anti-asthma effect than isoliquiritigenin suspensions	[58]
Chawla, et al.	Nano Micelles	Curcumin	Preclinical studies	Micellarcurcumin produces an anti-asthmatic effect in a sustained manner.	[61]
Sahib, et al.	Nano Micelles	Beclomethasonedipropionate	Preclinical studies	BAL fluid samples showed a marked decrease in the number of inflammatory cells.	[62]
Choi, et al.	Nano Micelles	Chil3 and Chil4 siRNA	In vitro	Asthma symptoms like airway inflammation and mucus production were reduced.	[63]
Onoue, et al.	Nano Micelles	Chafuroside A	Preclinical studies	Asthma may be treated with the self-assembled micellar formulation of chafuroside A.	[65]
Inapagolla, et al.	Dendrimer	Methylprednisolone	Preclinical studies	For the treatment of inflammatory illnesses such as asthma, dendrimer-conjugated drugs may improve medication retention in the lungs.	[60]
Mahanasr, et al.	Dendrimer	Beclomethasonedipropionate	Preclinical studies	Dexamethasone NPs are superior to free dexamethasone in reducing airway hyperresponsiveness.	[69]

## References

[1] Allan R., Canham K., Wallace R., Singh D., Ward J., Cooper A., Newcomb C. (2021). Usability and Robustness of the Wixela Inhub Dry Powder Inhaler. J. Aerosol Med. Pulm. Drug Deliv..

[2] Allen D.B. (2020). Inhaled Corticosteroids and Endocrine Effects in Childhood. Endocrinol. Metab. Clin. N. Am..

[3] Gautier C., Charpin D. (2017). Environmental triggers and avoidance in the management of asthma. J. Asthma Allergy.

[4] Shimoni S., Gendelman G., Ayzenberg O., Smirin N., Lysyansky P., Edri O., Deutsch L., Caspi A., Friedman Z. (2011). Differential effects of coronary artery stenosis on myocardial function: The value of myocardial strain analysis for the detection of coronary artery disease. J. Am. Soc. Echocardiogr..

[5] Ari A. (2021). A path to successful patient outcomes through aerosol drug delivery to children: A narrative review. Ann. Transl. Med..

[6] Louie S., Zeki A.A., Schivo M., Chan A.L., Yoneda K.Y., Avdalovic M., Morrissey B.M., Albertson T.E. (2013). The asthma-chronic obstructive pulmonary disease overlap syndrome: Pharmacotherapeutic considerations. Expert Rev. Clin. Pharmacol..

[7] Azizoglu E., Ozer O., Prausnitz M.R. (2022). Fabrication of pure-drug microneedles for delivery of montelukast sodium. Drug Deliv. Transl. Res..

[8] Belice P.J., Mosnaim G., Galant S., Kim Y., Shin H.W., Pires-Barracosa N., Hall J.P., Malik R., Becker E. (2020). The impact of caregiver health literacy on healthcare outcomes for low income minority children with asthma. J. Asthma.

[9] Biddiscombe M.F., Usmani O.S. (2021). Delivery and adherence with inhaled therapy in asthma. Minerva Med..

[10] Bridgeman M.B., Wilken L.A. (2021). Essential Role of Pharmacists in Asthma Care and Management. J. Pharm. Pract..

[11] Jeevanandam J., Barhoum A., Chan Y.S., Dufresne A., Danquah M.K. (2018). Review on nanoparticles and nanostructured materials: History, sources, toxicity and regulations. Beilstein. J. Nanotechnol..

[12] Brunaugh A.D., Sharma S., Smyth H. (2021). Inhaled fixed-dose combination powders for the treatment of respiratory infections. Expert Opin. Drug Deliv..

[13] Casula L., Sinico C., Valenti D., Pini E., Pireddu R., Schlich M., Lai F., Fadda A.M. (2021). Delivery of beclomethasone dipropionate nanosuspensions with an electronic cigarette. Int. J. Pharm..

[14] Cataldo D., Louis R., Michils A., Peché R., Pilette C., Schleich F., Ninane V., Hanon S. (2021). Severe asthma: Oral corticosteroid alternatives and the need for optimal referral pathways. J. Asthma.

[15] Emami F., Mostafavi S.J., Yazdi D.H. (2019). Na, Poly(lactic acid)/poly(lactic-co-glycolic acid) particulate carriers for pulmonary drug delivery. J. Pharm. Investig..

[16] Chen L., Arens R., Chidambaram A.G., Capponi S., Alshawa L., Claeys T.A., Hayes D., Robinson R.T. (2021). Vaping Associated Pulmonary Nontuberculous Mycobacteria. Lung.

[17] Cooper A., Parker J., Berry M., Wallace R., Ward J., Allan R. (2020). Wixela Inhub: Dosing Performance In Vitro and Inhaled Flow Rates in Healthy Subjects and Patients Compared with Advair Diskus. J. Aerosol Med. Pulm. Drug Deliv..

[18] Datusalia A.K., Singh G., Yadav N., Gaun S., Manik M., Singh R.K. (2021). Targeted delivery of montelukast for treatment of Alzheimer’s disease. CNS Neurol. Disord. Drug Targets.

[19] De Volder J., Vereecke L., Joos G., Maes T. (2020). Targeting neutrophils in asthma: A therapeutic opportunity?. Biochem. Pharmacol..

[20] Dhayanandamoorthy Y., Antoniraj M.G., Kandregula C.A.B., Kandasamy R. (2020). Aerosolized hyaluronic acid decorated, ferulic acid loaded chitosan nanoparticle: A promising asthma control strategy. Int. J. Pharm..

[21] Patil J.S., Sarasija S. (2012). Pulmonary drug delivery strategies: A concise, systematic review. Lung India Off. Organ Indian Chest Soc..

[22] Ashrafizadeh M., Zarrabi A., Hushmandi K., Zarrin V., Moghadam E.R., Hashemi F., Makvandi P., Samarghandian S., Khan H., Hashemi F. (2020). Toward Regulatory Effects of Curcumin on Transforming Growth Factor-Beta Across Different Diseases: A Review. Front. Pharmacol..

[23] Doroudian M., O’Neill A., Loughlin R.M., Prina-Mello A., Volkov Y., Donnelly S.C. (2021). Nanotechnology in pulmonary medicine. Curr. Opin. Pharmacol..

[24] Drmosh Q.A., Alade I.O., Qamar M., Akbar S. (2021). Zinc Oxide-Based Acetone Gas Sensors for Breath Analysis: A Review. Chemistry.

[25] Patra J.K., Das G., Fraceto L., Campos E., Rodríguez-Torres M.D.P., Acosta-Torres L., Diaz-Torres L., Grillo R., Swamy M., Sharma S. (2018). Nano based drug delivery systems: Recent developments and future prospects. J. Nanobiotechnol..

[26] Escarrer-Jaume M., Juliá-Benito J.C., Quevedo-Teruel S., del Prado A.P., Sandoval-Ruballos M., Quesada-Sequeira F., Álvaro-Lozano M. (2021). Changes in epidemiology and clinical practice in IgE-mediated Allergy in children. An. Pediatr..

[27] Fan P.S., Sun M.J., Qin D., Yuan C.S., Chen X.G., Liu Y. (2021). Nanosystems as curative platforms for allergic disorder management. J. Mater. Chem. B.

[28] Gulati N., Dua K., Dureja H. (2021). Role of chitosan based nanomedicines in the treatment of chronic respiratory diseases. Int. J. Biol. Macromol..

[29] Ren J., Liu Y., Yao Y., Feng L., Zhao X., Li Z., Yang L. (2021). Intranasal delivery of MSC-derived exosomes attenuates allergic asthma via expanding IL-10 producing lung interstitial macrophages in mice. Int. Immunopharmacol..

[30] Theoharides T.C. (2021). Ways to Address Perinatal Mast Cell Activation and Focal Brain Inflammation, including Response to SARS-CoV-2, in Autism Spectrum Disorder. J. Pers. Med..

[31] Honmane S., Hajare A., More H., Osmani R.A.M., Salunkhe S. (2019). Lung delivery of nanoliposomal salbutamol sulfate dry powder inhalation for facilitated asthma therapy. J. Liposome Res..

[32] Loureiro J.A., Andrade S., Ramalho M.J., Oliveira N., Pereira M.C. (2021). The interaction of a β2 adrenoceptor agonist drug with biomimetic cell membrane models: The case of terbutaline sulphate. Life Sci..

[33] Arafa M., Ayoub B. (2017). Nano-vesicles of salbutamol sulphate in metered dose inhalers: Formulation, characterization and in vitro evaluation. Int. J. App. Pharm..

[34] Komalla V., Allam V., Kwok P.C.L., Sheikholeslami B., Owen L., Jaffe A., Waters S.A., Mohammad S., Oliver B.G., Chen H. (2020). A phospholipid-based formulation for the treatment of airway inflammation in chronic respiratory diseases. Eur. J. Pharm. Biopharm..

[35] Chen X., Huang W., Wong B.C., Yin L., Wong Y.F., Xu M., Yang Z. (2012). Liposomes prolong the therapeutic effect of anti-asthmatic medication via pulmonary delivery. Int. J. Nanomed..

[36] Wong J.-Y., Ng Z.Y., Mehta M., Shukla S.D., Panneerselvam J., Madheswaran T., Gupta G., Negi P., Kumar P., Pillay V. (2020). Curcumin-loaded niosomes downregulate mRNA expression of pro-inflammatory markers involved in asthma: An in vitro study. Nanomedicine.

[37] Konduri K.S., Nandedkar S., Düzgünes N., Suzara V., Artwohl J., Bunte R., Gangadharam P.R. (2003). Efficacy of liposomal budesonide in experimental asthma. J. Allergy Clin. Immunol..

[38] Ng Z., Wong J.-Y., Panneerselvam J., Madheswaran T., Kumar P., Pillay V., Hsu A., Hansbro N., Bebawy M., Wark P. (2018). Assessing the Potential of Liposomes Loaded with Curcumin as a Therapeutic Intervention in Asthma. Colloids Surf. B Biointerfaces.

[39] Alberca-Custodio R.W., Faustino L.D., Gomes E., Nunes F.P.B., de Siqueira M.K., Labrada A., Almeida R.R., Câmara N.O.S., da Fonseca D.M., Russo M. (2020). Allergen-Specific Immunotherapy with Liposome Containing CpG-ODN in Murine Model of Asthma Relies on MyD88 Signaling in Dendritic Cells. Front. Immunol..

[40] Raju R.S., Kumar M.N.S., Kannan E., Ambhore N.S., Mulukutla S., Gowthamarajan K. (2015). Drug loaded liposomes of mesalamine incorporated into disease responsive microgels (Micro) for treating allergic asthma: An approach using smart drug delivery system. Eur. Respir. J..

[41] Alvarez M.J., Echechipía S., García B., Tabar A.I., Martín S., Rico P., Olaguibel J.M. (2002). Liposome-entrapped D. pteronyssinus vaccination in mild asthma patients: Effect of 1-year double-blind, placebo-controlled trial on inflammation, bronchial hyperresponsiveness and immediate and late bronchial responses to the allergen. Clin. Exp. Allergy J..

[42] Li Q., Zhan S., Liu Q., Su H., Dai X., Wang H., Beng H., Tan W. (2018). Preparation of a Sustained-Release Nebulized Aerosol of R-terbutaline Hydrochloride Liposome and Evaluation of Its Anti-asthmatic Effects via Pulmonary Delivery in Guinea Pigs. AAPS PharmSciTech.

[43] Arora M., Patel K., Choudhary P., Jain P., Malhotra M., Trivedi P. (2012). Liposome: A Novel Aerosol Carrier of Doxophylline in Treatment of Chronic Asthma & Chronic Obstructive Pulmonary Disease. J. Mol. Gen. Med..

[44] Elhissi A., Islam M.A., Arafat B., Taylor M., Ahmed W. (2010). Development and characterisation of freeze-dried liposomes containing two anti-asthma drugs. Micro Nano Lett..

[45] Paranjpe M., Müller-Goymann C.C. (2014). Nanoparticle-mediated pulmonary drug delivery: A review. Int. J. Mol. Sci..

[46] Nassimi M., Schleh C., Lauenstein H.D., Hussein R., Lübbers K., Pohlmann G., Switalla S., Sewald K., Müller M., Krug N. (2009). Low cytotoxicity of solid lipid nanoparticles in in vitro and ex vivo lung models. Inhal. Toxicol..

[47] Paranjpe M., Neuhaus V., Finke J.H., Richter C., Gothsch T., Kwade A., Büttgenbach S., Braun A., Müller-Goymann C.C. (2013). In vitro and ex vivo toxicological testing of sildenafil-loaded solid lipid nanoparticles. Inhal. Toxicol..

[48] Wang W., Zhu R., Xie Q., Li A., Xiao Y., Li K., Liu H., Cui D., Chen Y., Wang S. (2012). Enhanced bioavailability and efficiency of curcumin for the treatment of asthma by its formulation in solid lipid nanoparticles. Int. J. Nanomed..

[49] Lv C., Li H., Cui H., Bi Q., Wang M. (2021). Solid lipid nanoparticle delivery of rhynchophylline enhanced the efficiency of allergic asthma treatment via the upregulation of suppressor of cytokine signaling 1 by repressing the p38 signaling pathway. Bioengineered.

[50] Da Silva A.L., Martini S.V., Abreu S.C., Cdos S.S., Diaz B.L., Fernezlian S., de Sá V.K., Capelozzi V.L., Boylan N.J., Goya R.G. (2014). DNA nanoparticle-mediated thymulin gene therapy prevents airway remodeling in experimental allergic asthma. J. Control Release.

[51] Oyarzun-Ampuero F.A., Brea J., Loza M.I., Torres D., Alonso M.J. (2009). Chitosan-hyaluronic acid nanoparticles loaded with heparin for the treatment of asthma. Int. J. Pharm..

[52] Chattopadhyay P., Pathak M., Patowary P., Chakrabarti S., Goyary D., Karmakar S., Dwivedi S. (2020). Synthesized atropine nanoparticles ameliorate airway hyperreactivity and remodeling in a murine model of chronic asthma. J. Drug Deliv. Sci. Technol..

[53] Yong D., Saker S., Chellappan D.K., Madheswaran T., Panneerselvam J., Wadhwa R., Kumar P., Pillay V., Gupta G., Oliver B. (2019). Preparation, characterization and in-vitro efficacy of quercetin loaded liquid crystalline nanoparticles for the treatment of asthma. J. Drug Deliv. Sci. Technol..

[54] Ramelli S.C., Comer B.S., McLendon J.M., Sandy L.L., Ferretti A.P., Barrington R., Sparks J., Matar M., Fewell J., Gerthoffer W.T. (2020). Nanoparticle Delivery of Anti-inflammatory LNA Oligonucleotides Prevents Airway Inflammation in a HDM Model of Asthma. Mol. Ther. Nucleic Acids.

[55] Wang D., Nasab E., Athari S. (2021). Study effect of Chitosan-nanoparticle encapsulated/loaded Baicalein on allergic Asthma pathology in mouse model. Saudi J. Biol. Sci..

[56] Wang K., Feng Y., Li S., Li W., Chen X., Yi R., Zhang H., Hong Z. (2018). Oral Delivery of Bavachinin-Loaded PEG-PLGA Nanoparticles for Asthma Treatment in a Murine Model. J. Biomed. Nanotechnol..

[57] Chakraborty S., Ehsan I., Mukherjee B., Mondal L., Roy S., Saha K.D., Paul B., Debnath M., Bera T. (2019). Therapeutic potential of andrographolide-loaded nanoparticles on a murine asthma model. Nanomed. Nanotechnol. Biol. Med..

[58] Cao M., Zhan M., Wang Z., Wang Z., Li X.-M., Miao M. (2020). Development of an Orally Bioavailable Isoliquiritigenin Self-Nanoemulsifying Drug Delivery System to Effectively Treat Ovalbumin-Induced Asthma. Int. J. Nanomed..

[59] Casula L., Lai F., Pini E., Valenti D., Sinico C., Cardia M.C., Marceddu S., Ailuno G., Fadda A.M. (2021). Pulmonary Delivery of Curcumin and Beclomethasone Dipropionate in a Multicomponent Nanosuspension for the Treatment of Bronchial Asthma. Pharmaceutics.

[60] Inapagolla R., Guru B., Emre Y., Gao X., Lieh-Lai M., Bassett D.J.P., Kannan R.M. (2010). In vivo efficacy of dendrimer-methylprednisolone conjugate formulation for the treatment of lung inflammation. Int. J. Pharm..

[61] Chawla R., Sahu B., Mishra M., Rani V., Singh R. (2022). Intranasal micellar curcumin for the treatment of chronic asthma. J. Drug Deliv. Sci. Technol..

[62] Sahib M., Darwis Y., Kok-Khiang P., Abdulameer S., Tan Y. (2012). Incorporation of Beclomethasone Dipropionate into Polyethylene Glycol-Diacyl Lipid Micelles as a Pulmonary Delivery System. Drug Dev. Res..

[63] Choi M., Jeong H., Kim S., Kim M., Lee M., Rhim T. (2019). Targeted delivery of Chil3/Chil4 siRNA to alveolar macrophages using ternary complexes composed of HMG and oligoarginine micelles. Nanoscale.

[64] Wadhwa R., Shukla S.D., Chellappan D.K., Gupta G., Collet T., Hansbro N., Oliver B., Williams K., Hansbro P.M., Dua K. (2019). Phytotherapy in Inflammatory Lung Diseases: An Emerging Therapeutic Interventional Approach. Phytochemistry: An In-Silico and In-Vitro Update.

[65] Onoue S., Matsui T., Aoki Y., Ishida H., Nukaya H., Kou K., Yamada S. (2011). Self-assembled micellar formulation of chafuroside A with improved anti-inflammatory effects in experimental asthma/COPD-model rats. Eur. J. Pharm. Sci..

[66] Paudel K.R., Mehta M., Yin G.H.S., Yen L.L., Malyla V., Patel V.K., Panneerselvam J., Madheswaran T., MacLoughlin R., Jha N.K. (2022). Berberine-Loaded Liquid Crystalline Nanoparticles Inhibit Non-Small Cell Lung Cancer Proliferation and Migration in Vitro. Environ. Sci. Pollut. Res..

[67] Wadhwa R., Aggarwal T., Thapliyal N., Chellappan D.K., Gupta G., Gulati M., Collet T., Oliver B., Williams K., Hansbro P.M. (2019). Nanoparticle-based drug delivery for chronic obstructive pulmonary disorder and asthma: Progress and challenges. Nanotechnology in Modern Animal Biotechnology.

[68] Vij N., Min T., Bodas M., Gorde A., Roy I. (2016). Neutrophil Targeted Nano-Drug Delivery System for Chronic Obstructive Lung Diseases. Nanomed. Nanotechnol. Biol. Med..

[69] Nasr M., Najlah M., D’Emanuele A., Elhissi A. (2013). PAMAM Dendrimers as Aerosol Drug Nanocarriers for Pulmonary Delivery Via Nebulization. Int. J. Pharm..

[70] Pandey P., Purohit D., Chellappan D.K., Gupta G., Tambuwala M.M., Aljabali A.A., Satija S., Dureja H. (2020). Advancement in translational respiratory research using nanotechnology. Targeting Chronic Inflammatory Lung Diseases Using Advanced Drug Delivery Systems.

[71] Patil A., Kydarkunte A., Patole M., Pokharkar V. (2014). Montelukast-loaded nanostructured lipid carriers: Part II Pulmonary drug delivery and in vitro–in vivo aerosol performance. Eur. J. Pharm. Biopharm..

[72] Gupta G., Chellappan D.K., Singh S.K., Gupta P.K., Kesari K.K., Jha N.K., Thangavelu L., Oliver B.G., Dua K. (2021). Advanced drug delivery approaches in managing TGF-β-mediated remodeling in lung diseases. Nanomedicine.

[73] Mehrabi Nasab D., Taheri A., Athari S.S. (2020). Design and Fabrication of Gold Nanoparticles for Anti-Asthma Drug Delivery. Arch. Med. Lab. Sci..

[74] Omlor A., Le D., Schlicker J., Hannig M., Ewen R., Heck S., Herr C., Kraegeloh A., Hein C., Kautenburger R. (2016). Local Effects on Airway Inflammation and Systemic Uptake of 5 nm PEGylated and Citrated Gold Nanoparticles in Asthmatic Mice. Small.

[75] Chan Y., Allam V.S.R.R., Paudel K.R., Singh S.K., Gulati M., Dhanasekaran M., Gupta P.K., Jha N.K., Devkota H.P., Gupta G. (2021). Nutraceuticals: Unlocking newer paradigms in the mitigation of inflammatory lung diseases. Crit. Rev. Food Sci. Nutr..

[76] Park H., Kim K., Jang S., Park J.w., Cha H., Lee J., Kim J.-O., Kim S., Lee C., Kim J. (2010). Attenuation of allergic airway inflammation and hyperresponsiveness in a murine model of asthma by silver nanoparticles. Int. J. Nanomed..

[77] Chan Y., Mehta M., Paudel K.R., Madheswaran T., Panneerselvam J., Gupta G., Su Q.P., Hansbro P.M., MacLoughlin R., Dua K. (2021). Versatility of liquid crystalline nanoparticles in inflammatory lung diseases. Nanomedicine.

[78] Valluri V., Katari N., Khatri C., Vyas G., Polagani S. (2021). Highly sensitive liquid chromatography–tandem mass spectrometry assay for the determination of azathioprine in presence of mercaptopurine and its application to a human pharmacokinetic study. Sep. Sci. Plus.

[79] Halwani R., Shaik A., Ratemi E., Afzal S., Kenana R., Al-Muhsen S., al Faraj A. (2016). A novel anti-IL4R nanoparticle efficiently controls lung inflammation during asthma. Exp. Mol. Med..

[80] Wu Y., Shi W., Wang H., Yue J., Mao Y., Zhou W., Kong X., Guo Q., Zhang L., Xu P. (2020). Anti-ST2 Nanoparticle Alleviates Lung Inflammation by Targeting ILC2s-CD4(+)T Response. Int. J. Nanomedicine.

[81] Anderson C., Grimmett M., Domalewski C., Cui H. (2019). Inhalable nanotherapeutics to improve treatment efficacy for common lung diseases. Nanomed. Nanobiotechnol..

[82] Vyas G., Mathur M., Patel N.A., Patel R.P. (2017). Aphrodisiac Efficacy of Blepharis sindica seeds: A comparative assessment using different solvent types. Indian J. Biochem. Biophys..

[83] Mathur M., Vyas G. (2013). Role of nanoparticles for production of smart herbal drug-An overview. Indian J. Nat. Prod. Resour..

[84] Zhao M.-Z., Li Y., Han H.-Y., Lihua M., Yang G., Liu Z.-Q., Ma C., Yang P.-C., Liu S. (2020). Specific Ag-guiding nano-vaccines attenuate neutrophil-dominant allergic asthma. Mol. Immunol..

[85] Subramanian V.B., Katari N.K., Ponnam V., Konduru N., Dongala T., Marisetti V.M., Vyas G. (2021). Stability-indicating reversed-phase-HPLC method development and validation for sacubitril/valsartan complex in the presence of impurities and degradation products: Robustness by quality-by-design approach. Biomed. Chromatogr..

[86] Conde E., Bertrand R., Balbino B., Bonnefoy J., Stackowicz J., Caillot N., Colaone F., Hamdi S., Houmadi R., Loste A. (2021). Dual vaccination against IL-4 and IL-13 protects against chronic allergic asthma in mice. Nat. Commun..

[87] Chan Y., Ng S.W., Chellappan D.K., Madheswaran T., Zeeshan F., Kumar P., Pillay V., Gupta G., Wadhwa R., Mehta M. (2021). Celastrol-loaded liquid crystalline nanoparticles as an anti-inflammatory intervention for the treatment of asthma. Int. J. Polym. Mater. Polym. Biomater..

[88] Grozdanovic M., Laffey K., Abdelkarim H., Hitchinson B., Harijith A., Moon H.G., Park G., Rousslang L., Masterson J., Furuta G. (2018). Novel Peptide Nanoparticle Biased Antagonist of CCR3 Blocks Eosinophil Recruitment and Airway Hyperresponsiveness. J. Allergy Clin. Immunol..

[89] Kumar M., Kong X., Behera A., Hellermann G., Lockey R., Mohapat S. (2003). Chitosan IFN-gamma-pDNA nanoparticle (CIN) therapy for allergic asthma. Genet. Vaccines Ther..

[90] Kong X., Hellermann G., Zhang W., Jena P., Kumar M., Behera A., Behera S., Lockey R., Mohapat S. (2008). Chitosan Interferon-γ Nanogene Therapy for Lung Disease: Modulation of T-Cell and Dendritic Cell Immune Responses. Allergy Asthma Clin. Immunol..

[91] Muchakayala S.K., Katari N.K., Dongala T., Marisetti V.M., Vyas G., Vegesna R.V.K. (2021). Eco-friendly and green chromatographic method for the simultaneous determination of chlorocresol and betamethasone dipropionate in topical formulations using Box–Behnken design. J. Iran. Chem. Soc..

